# Probability, parameters, and duration of immigration and extinction in microbial communities

**DOI:** 10.1093/ismejo/wrag195

**Published:** 2026-07-22

**Authors:** Thomas P Curtis, Ben Allen, Mathew Brown, Amy Bell, Donna Swan, Russell Davenport, William Sloan

**Affiliations:** School of Engineering, Newcastle University, Newcastle NE1 7RU, United Kingdom; School of Engineering, Newcastle University, Newcastle NE1 7RU, United Kingdom; School of Engineering, Newcastle University, Newcastle NE1 7RU, United Kingdom; School of Engineering, Newcastle University, Newcastle NE1 7RU, United Kingdom; School of Engineering, Newcastle University, Newcastle NE1 7RU, United Kingdom; School of Engineering, Newcastle University, Newcastle NE1 7RU, United Kingdom; Department of Civil Engineering, Glasgow University, Glasgow G12 8QQ, United Kingdom

**Keywords:** microbes, immigration, gambler’s ruin, communities, wastewater, infection, extinction

## Abstract

We propose a suite of simple equations to estimate the probability and duration of two important processes in microbial ecology: immigration and extinction. Our work is based on the gambler’s ruin equation, which determines the probability that a number of immigrants (*i*) can attain an abundance *N* given the ratio of the probabilities of death *q* and division (or birth) *p*. We estimate the probability of an organism attaining a value of *N* in the context of bioaugmentation, transplantation, infection, mutation, and extinction. For example, an inoculum of 10^8^ bacteria with a *q/p* of 1.00000001 has a 10^−43^ chance of attaining an abundance of 10^10^. The ratio of deaths to births controls the immigration parameter used in neutral models (*m*), and infectious dose in pathogens. We use *Vibrio cholerae* infections to demonstrate that the gambler’s ruin equation can be used to estimate the infectious dose in naturally occurring infections. We calculated the long-term average value of *m* and *q/p* in a wastewater treatment plant*.* All values of *q/p* were ≥1. We expect the long-term average value of *q/p* to be ~1 in all stable microbial communities. In the absence of migration, bacterial populations with *q/p* ≥ 1 will go extinct with probability 1. We use the ratio *q/p* and simple recurrence relationships to estimate the time for a given change in abundance to occur. When *q/p* = 1, extinction in even a small microbial population will take thousands of years. Our simple mechanistic models could play a powerful role in theory and practice.

## Introduction

In this paper, we consider simple models and associated parameters for the fate of a bacterium or bacteria of a given species when introduced into or lost from a complex microbial community. The arrival of new individuals is a feature of virtually all natural microbial communities and is called immigration. Immigration is of both intrinsic and practical importance. Ecologists use the term “invasion” when the immigrant is novel. Extinction, the loss of a species, is also of fundamental importance. Practical examples of extinction include recovery from infection and the fate of a genetically manipulated organism in the environment. Because extinction is simply the reverse of invasion, it can, in principle, be described by the same simple models as immigration. The possibility of capturing these phenomena within a common mathematical framework is, in part, obscured by the wide range of terms used in the literature.

In ecology and microbial ecology, the terminology is already established. The number of immigrants is sometimes referred to as “propagule pressure”, a term often used qualitatively. However, the probability of establishing a growing immigrant population has been quantitatively related to the number of immigrants, with varying degrees of complexity [1, 2]. The effect of immigration on community diversity has been quantitatively related to immigration in simple birth-death models [3, 4]. In such models, the probability that a death in a community is replaced by an immigrant is expressed as *m*. Numbers are assumed to remain constant, so the probability of a death being replaced by growth (sometimes called a birth) is therefore 1 − *m*.

The environmental engineering and environmental biotechnology communities sometimes propose “engineering” immigration by adding bacteria to treat waste, a process called bioaugmentation. Bioaugmentation does not always succeed in treating a given waste, and we lack both criteria for success and methods to predict it. In wastewater treatment, where immigration occurs “naturally” via the influent wastewater, engineers use other definitions, including “mass migration”, and “net growth”. Mass migration is the proportion of the reactor community derived from the influent [5]. Net growth is calculated from the difference between growth and immigration, and biomass wastage and, in principle, other losses [6–8].

In therapeutic settings, some practitioners will propose manipulating immigration by adding organisms, a process often referred to as probiotic therapy. In faecal transplantation, whole communities may be administered to a patient. In both cases, clear criteria for success and methods to predict it might be valuable [9].

In human, animal, and plant pathology, immigration by a pathogen initiates an infection. The ability of a pathogen to infect is expressed as the 50% infectious dose (ID_50_), the number of microorganisms required to cause disease in half of the exposed population. ID_50_ values are determined experimentally from human or animal experiments [10, 11]. Quantitative studies of infection led Meynell to propose [12, 13] that each invading bacterium has the potential to cause an infection (“the hypothesis of independent action”). Several researchers drew on Kendall [14] to place this idea on a solid theoretical foundation, using a suite of stochastic death/growth models [15–17]. However, these models were difficult to calibrate because they treated the probabilities of birth and death independently [18]. Some authors noted that the ID_50_ could be inferred from the ratio of deaths to births, if the number of pathogens in an infected subject were assumed to be very high [18, 19].

Researchers and policymakers developing tools to predict the risk of infection from environmental exposure to enteric pathogens drew on the hypothesis of independent action. They used this work and empirical analyses of dose–response curves to estimate the probability of infection in hypothetical scenarios. A procedure now known as quantitative microbial risk assessment (QMRA) [20]. Unfortunately, some of the dose–response curves were derived from human experiments on male prisoners undertaken in the 1960s. It is unclear whether those experiments would meet today’s ethical standards. Moreover, such experiments may not reflect the behaviour of a pathogen in a community where all members are exposed. Obtaining scientifically and ethically acceptable infectivity data is a challenge for those using QMRA.

In this manuscript, we (re)introduce the use of the ratio of deaths to births in the simple gambler’s ruin equation [21] (p. 213). We then: (i) present straightforward numerical illustrations of how the equation predicts the probabilities of immigration and extinction in two simple scenarios; (ii) show how to estimate the key parameter (the ratio of deaths to births) in two natural contexts; and (iii) use that ratio to estimate the time required for immigration or extinction to occur.

## Materials and methods

The methods are described in the supplementary information and were previously reported [22]. Briefly, grab samples were taken from the influent and mixed liquor of a full-scale nitrifying activated sludge plant. Samples were collected weekly for 5 years (June 2011 to June 2016; 258 sampling events). DNA was extracted from 250 μl of mixed liquor and from 15 ml of influent. Sample preparation for sequencing generally followed the protocol of Caporaso et al. (2012), and the V4 region of the bacterial 16S rRNA gene was amplified. Quantification of total bacteria was performed using qPCR targeting the 16S rRNA gene. Samples were amplified in triplicate on a CFX96 Real-Time PCR Detection System (Bio-Rad, UK) using the primer sets 338F [23] and 1046R [24]. Amplicon sequence variant (ASV) absolute abundances per sample were calculated by multiplying ASV relative abundances by accompanying total bacteria numbers. All equations used in the paper are derived in the appendices and relevant code is available at https://github.com/beadyallen/PPD_Immigration_Microbe.

## Results

### The model

If we observe a microbe long enough, it will do one of two things: reproduce or die. The probability of reproduction (or “birth”) is *p,* and the probability of death is *q*. The probability of doing neither is 1-*p*-*q*, which is entirely defined by the other two probabilities. In this model, 1-*p-q* = 0. We are interested in whether the microbe’s descendants ultimately attain an abundance of *N* or, alternatively, go extinct. The chosen abundance *N* can be an arbitrary parameter, an engineering goal, or the carrying capacity (often represented by *K* in ecological models [25]).

This is a classic scenario in probability and Markov chains, often known as the gambler’s ruin problem. It is so called because it is like entering a casino with *i* francs (Pascal is credited with solving the problem) and playing a game with probabilities *p* and *q* of winning or losing a franc, respectively. A solution can be found in Appendix 1 and in classical texts [21] (p. 213).

Let ${P}_i$ be the probability that a population starting with *i* individuals will eventually reach *N*. If *q*/*p* is not equal to 1, that is, if the probability of a single organism doubling is greater than or less than the probability of it dying, then:


(1) \begin{equation*} {P}_i=\left(\frac{1-{\left(\frac{q}{p}\right)}^i}{1-{\left(\frac{q}{p}\right)}^N}\right) \end{equation*}

If *q/p* is 1


(2) \begin{equation*} {P}_i=\frac{\ i}{N} \end{equation*}

Thus, if birth is more likely than death, the probability of successful immigration is higher. If the ratio of deaths to births exceeds 1, successful immigration becomes unlikely. We assume that random (Figure A3) or systematic differences in the ratio of deaths to births are not too important. An interesting corollary is that if *q/p* ≥ 1 and the gambler plays forever (equivalent to setting *N* to infinity), the probability of extinction will be 1.

Superficially, it appears to be a simple binary: if *q/p* is less than 1, immigration will occur; if it is greater than 1, it will not. But for values close to 1 (which, as we will show, are the norm), a subtler picture emerges.

These subtleties become apparent when we examine the model in separate contexts: mutation, bioaugmentation, continuous-flow systems, and infection and use the ratio of deaths to births to estimate the time required for a change to occur.

### Mutation, where *i* = 1

**Figure 1:**
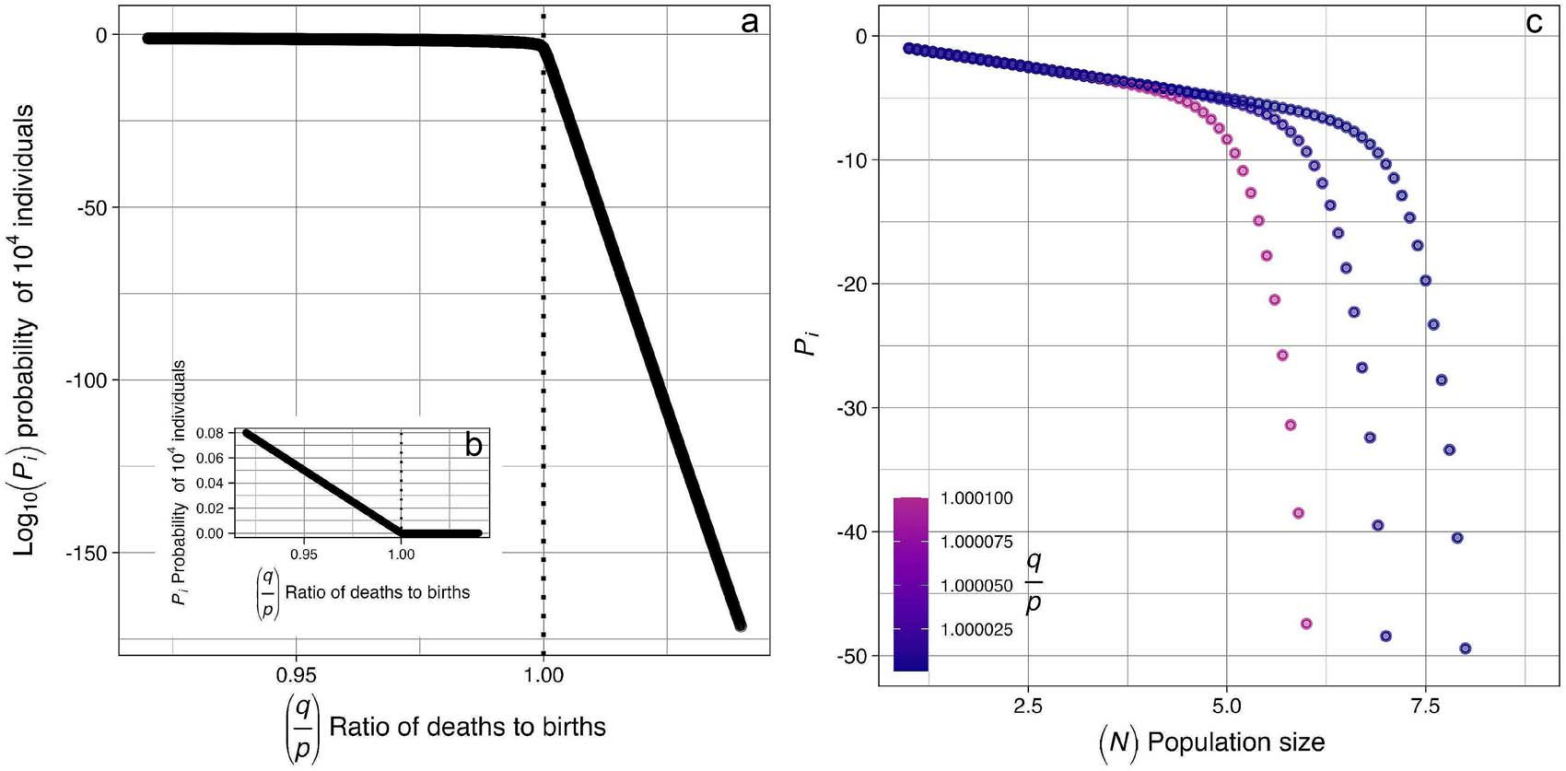
The fate of a single organism at different ratios of death to birth. (a) The probability of a single bacterium (*i* = 1) achieving an abundance of 10^4^ (*N* = 10^4^) across a plausible range of ratios of death to growth. (b) The inset is a non-logarithmic version of 1A in the region where *q/p* ~ 1. (c) The log_10_ probability (*P_i_*) of a single immigrant or mutant (*i* = 1) with a slight disadvantage (*q/p* = 1.0001) attaining an abundance *N_i_.*

Consider the case of *i* equal to 1 (Figure 1). That is, there is just one immigrant. This is probably an extremely common event. While it is unlikely that anyone would add a single bacterium to an environment, a mutant will inevitably arise in ~1/300 births [26]. Because most mutations are unique, this is equivalent to immigration by a single novel organism. In a system with 10^18^ individuals and a growth rate of 0.1 d^−1^, there will be ~3 × 10^14^ such events per day. With so many events, “low-probability” events can be of interest. Thus, it is quite possible that a fraction of slightly disadvantaged (~*q*/*p* < 1.0001) bacteria generated each day by mutation can attain a modest abundance.

In larger communities (10^13^–10^18^ bacteria), there may always be a substantial background of mutations ready for selection should circumstances allow. The modest abundance they attain by chance could reduce the time required for them to become abundant and thus of functional importance in a system. More disadvantaged mutations (*q*/*p* > 1.05) are unlikely to attain even a modest abundance. For example, a single mutation with a *q*/*p* of 1.05 has less than a 10^−21^ chance of achieving an abundance of 10^3^. Conversely, an organism with a modest advantage may go extinct before attaining even a modest abundance (Figure 1b). Thus, a single organism with a *q/p* of 0.95 is ~20 times more likely to go extinct than to attain an abundance of 10^4^.

### Probiotics and bioaugmentation where *i* > 1

In therapeutics, environmental biotechnology, and environmental engineering, we may wish to add bacteria to a community in the hope that they will reach sufficient abundance to positively impact the ecosystem. However, a practitioner proposing such a course of action will wish to have a high (say 90%) chance of success. Success here means becoming sufficiently abundant to have the desired impact on the system. Success is context specific. However, we will assume success requires forming 0.1% of the biomass. In a biological treatment plant with 10^18^ individuals, this would imply *N* = 10^15^_._ It is essentially impossible for an organism with a *q*/*p* value of >1 to attain a meaningful abundance. For example, an inoculum of 10^8^ bacteria with a *q*/*p* of 1.00000001 has a 10^−43^ chance of attaining an abundance of 10^10^.

Where death and growth match perfectly, *q*/*p* is exactly 1. We can therefore use Equation 2 to deduce that:


(3) \begin{equation*} i={P}_iN \end{equation*}

So, to achieve a 90% chance of success (*P_i_* = 0.9), the inoculum (*i*) must be 90% of the desired final abundance (*N*), a proportion more akin to a transplantation than bio-augmentation.

**Figure 2:**
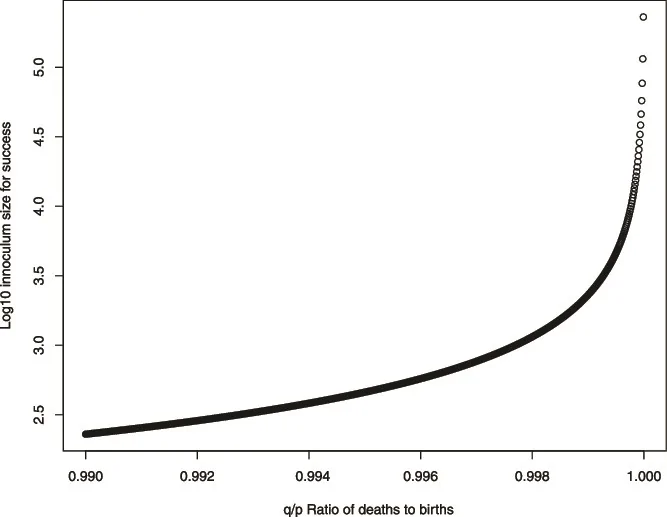
The inoculum required for a 90% chance of success at a given ratio (*q/p*) of deaths to births, assuming the desired abundance, *N,* is 10^6^. The figure is truncated at a *q/p* of 0.99; however, the required inoculum continues to decrease substantially as *q/p* falls below this value.

When *q/p* is less than 1, success is not assured, but the dose required for a 90% chance of success can be estimated (Figure 2) by rearranging equation 1 and setting *P_i_* to the target probability.


(4) \begin{equation*} i=\frac{\mathit{\ln}\left({P}_i{\left(\frac{q}{p}\right)}^N+1-{P}_i\right)}{\mathit{\ln}\left(\frac{q}{p}\right)}\kern1.25em \end{equation*}

The desired ratio or inoculum can now be calculated using equation 4. In principle, practitioners could also increase their chances of success and reduce the required inoculum by manipulating the environment to lower the ratio of deaths to births (Figure 2).

### Estimating the value of *q*/*p* in an engineered biological system and in microbial ecology more generally

In microbial ecology, immigration is denoted by *m*, the probability that an immigrant replaces a death. Typically, *m* has been inferred by fitting a model to data [4, 27] as the compound parameter *N_T_m*, where *N_T_* is the total number of bacteria in the system. Recently, we [28] demonstrated that *m* could be calculated directly in a chemostat. We have extended this reasoning to the aeration basin of a full-scale wastewater treatment plant and, in doing so, show a simple relationship between *m* and the parameter $\frac{q}{p}$.

The change in the number of a particular type of bacteria in a system is simply the sum of the number entering, the number dying (or leaving), and the number growing. If *X* is the abundance of a particular bacterial species (cells/m^3^) (*X*_inf_ and *X*_reactor_, the abundance in the influent and mixed liquor, respectively), *Q* is the flow (m^3^/day) of influent entering the reactor, *μ* is the bacteria’s growth rate (d^−1^), *V* is the volume (m^3^) of the reactor and *θ* is the fraction of bacteria dying per day due to sludge wastage and other causes (d^−1^), then:


(5) \begin{equation*} \frac{dXV}{dt}={X}_{\mathrm{inf}}Q+\mu{X}_{\mathrm{reactor}}V-\theta{X}_{\mathrm{reactor}}V \end{equation*}

At steady state, with *μ* ≥ 0, and noting that hydraulic retention time (HRT, d) is *V/Q,* we can rearrange equation 5 (see Appendix 2) to express the parameters per death, yielding:


(6) \begin{equation*} 1=\frac{X_{\mathrm{inf}}}{{\theta X}_{\mathrm{reactor}}\mathrm{HRT}}+\frac{\mu }{\theta } \end{equation*}

The parameter *m* is the number of immigrations per death, so at steady state:


(7) \begin{equation*} m=1-\frac{\mu }{\theta }=\frac{X_{inf}}{{\theta X}_{\mathrm{reactor}}\mathrm{HRT}} \end{equation*}

This equation shows the relationship between immigration, growth, biomass death in the reactor, and the effluent and reactor conditions. Thus, we have an equilibrium relationship between the immigration parameter *m* and the ratio *q/p*:


(8) \begin{equation*} \frac{q}{p}\sim \frac{\theta }{\mu }=\frac{1}{1-m} \end{equation*}

The term *m* is a probability, so it cannot exceed 1. When *m = 1,* all deaths are replaced by immigration. This occurs when *X*_inf_/*X*_reactor_ *= 1/*HRT*θ. At ratios below this, it is no longer possible to replace all deaths with immigration, and so the assumptions of the model are violated. When *X*_inf_/*X*_reactor_ = 1/HRT*θ, the mean net growth rate of Saunders [8] is zero, so an *m* value of ≤1 corresponds to a growth rate ≥ 0. The value of *m* can be determined directly from the influent and mixed-liquor bacterial concentrations.

In a study described previously [22], the number and diversity of bacteria in the influent and mixed liquor of an activated sludge plant were monitored weekly for 2 years. Biomass concentrations measured using gravimetric (volatile suspended solids, or VSS) and molecular (qPCR of the 16S gene) methods were stationary (Augmented Dickey-Fuller test, *P* < .01 for a lag of 6 in all cases). Stationarity implies that the mean, variance, and covariance do not change over time. The mean sludge age in that period was 11.58 days, implying a death rate of 0.1, and the HRT was 0.5 days. On this basis, we can calculate the values of *m* and thus *q/p* for all taxa with a ratio of *X*_inf_/*X*_reactor_ of >0.043.

By quantifying total bacterial abundance in the influent wastewater and the reactor, and by knowing the sludge age and HRT, we can estimate *m* for many taxa. The estimates of *m* are potentially subject to systematic errors due to differences in DNA extraction efficiency between settled sewage and activated sludge and from under- or overestimating deaths (see Appendix 5). However, such errors will shift the entire data set up or down, keeping the relative values constant.

**Figure 3:**
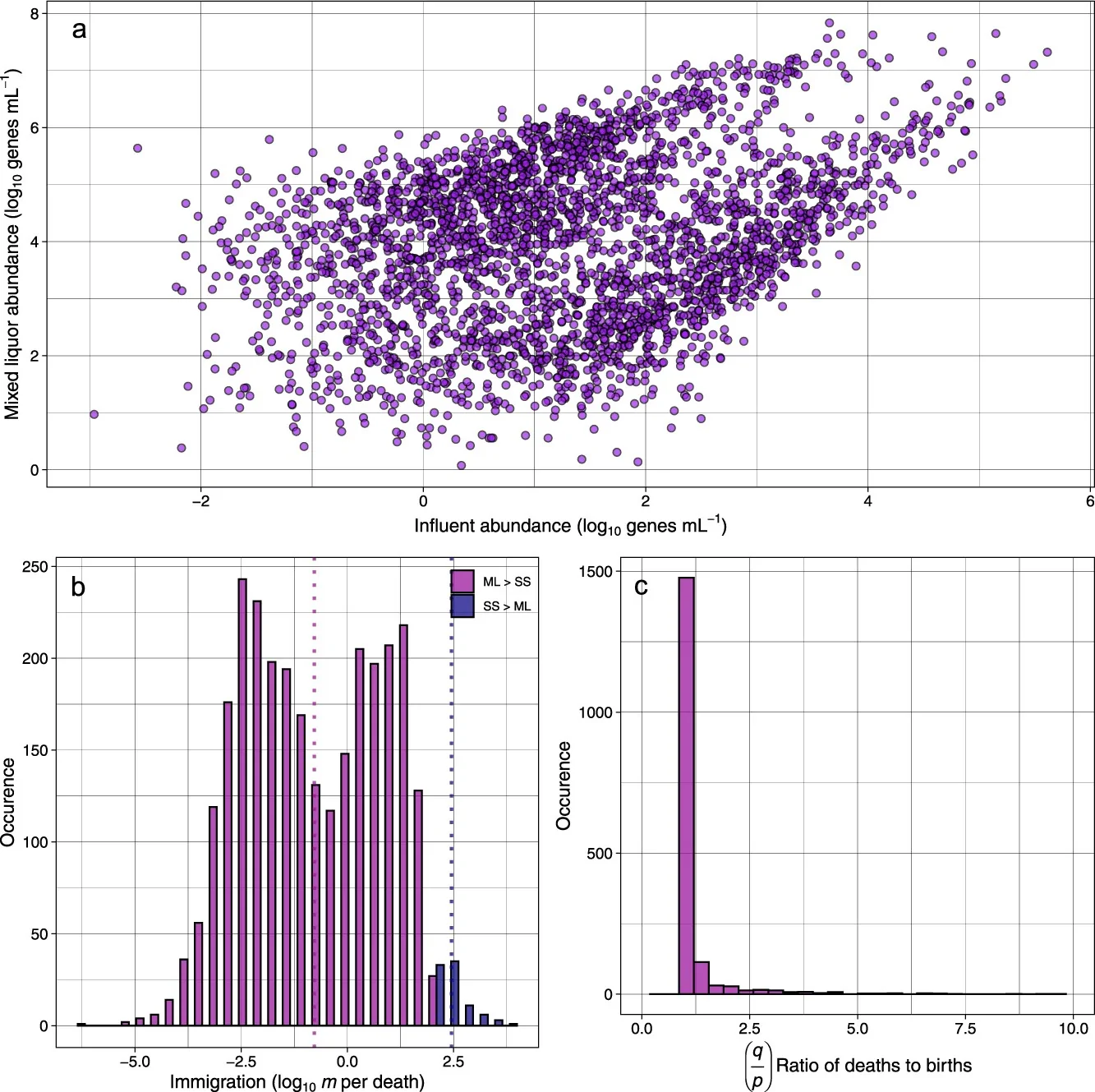
(a) Comparison of ASV abundances in the influent and mixed liquor of the activated sludge plant. (b) The bimodal distribution of immigration rates averaged over 5 years in an activated sludge plant. Values of *m* > 1 violate the assumptions of the method because net growth rates fall below zero. The data are shown for all taxa detectable in both samples, as systematic errors introduce uncertainty about the absolute, but not the relative, values of m for these organisms. SS, settled sewage; ML, mixed liquor. The dotted line represents the mean values for *m* for the taxa more abundant in the mixed liquor than settled sewage (left) and vice versa (right). (c) The distribution of the ratio of deaths to births in activated sludge.

**Figure 4:**
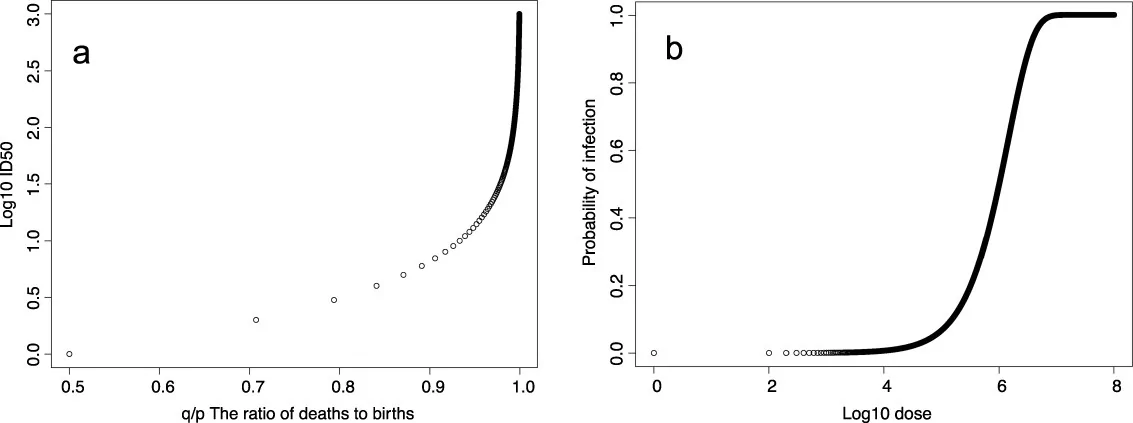
(a) The relationship between the ratio of deaths to births and the probability of infection (the actual values are less than 0.99999). (b) A theoretical dose abundance curve for *Salmonella*, assuming a ratio of deaths to births of 0.99999931.

The range of values is large (~7 orders of magnitude) and bimodal (Figure 3a and b). The taxa fall into two groups: those relatively more abundant in the influent but less abundant in the reactor, and vice versa (Figure 4a). Because the probability of replacement by growth is 1 − *m*, any bacterium with an *m* value <1 is growing in the reactor.

Many taxa will be overlooked because detection limits will impose upper and lower bounds on the observed values of *m*. However, only 0.8% of the reads in the settled sewage were from taxa found exclusively in the settled sewage, and 1.2% of the reads in the mixed liquor were found exclusively in the mixed liquor.

Take, e.g. the genus *Nitrosomonas*. Of the 17 ASVs detected, 7 were found only in the activated sludge, and 4 were found only in the settled sewage. However, they accounted for just 3.2% and 0.6% of the *Nitrosomonas* biomass in the activated sludge and settled sewage, respectively. The overall *m* value for the genus was 0.0014, implying ~690 births per immigration event.

Using equation 8, we can see that the values of *q/p* are at, or very close to, 1 (Figure 3c). A value of 1 was returned when a taxon was not detected in the influent. Such an organism accounted for only 0.8% of the mixed liquor biomass. For the remainder, the smallest detectable ratio was 1.000000665 (from the family *Gemmatimonadaceae*). Only 8% of taxa had a *q/p* ratio > 10.

### Estimating the value of *q*/*p* in infections and estimating infectivity

A pathogen is an organism with *q/p* < 1 [13, 15]. Moreover, if a subject is made ill or killed by an infection, the number of organisms infecting them will be very large. When *q/p* is <1 and *N* >> 1, the denominator in equation 1 quickly approximates to 1, and the gambler’s ruin equation simplifies to the textbook relationships between the probability of infection and dose [13]:


(9) \begin{equation*} {P}_i=1-{\left(\frac{q}{p}\right)}^i\kern1em \end{equation*}

Thus, if we know the infectious dose required to infect 50% of subjects (ID_50_), we can calculate *q*/*p*:


(10) \begin{equation*} {0.5}^{1/i}=\left(\frac{q}{p}\right)\kern1em \end{equation*}

Which is an alternative to Shortley and Wilkins’ equation (*q/p* = 1 − (0.69/ID_50_).

On this basis, one can calculate the relationship between *q*/*p* and ID_50_ (Figure 4a) and, conversely, the ratio of deaths to births from ID_50_. The ratio is close to, but less than 1. Thus, in mice, *Campylobacter* serotype PEN 2 has an ID_50_ of 6.68 × 10^4^ [29, 30], suggesting a *q*/*p* of 0.9999896. In humans, *Salmonellae* have an ID_50_ of ~10^6^ [31] [10], implying a *q*/*p* of 0.99999931. Classical toxigenic *Vibrio cholerae* has an ID_50_ of ~10^8^ [10], suggesting that *q*/*p* is 0.9999999931. Clearly, small changes in either deaths or births could be significant for infection.

Equation 9 (*P* = 1 − (*q*/*p*)*^i^*) can be used to construct a plausible dose–response curve (Figure 4b) and to inform microbial risk assessment (usually expressed as *P* = 1 − *e*^−*ki*^ where *k* is a fitted parameter) [32]. The fitted parameter *k* has a simple relationship with the theoretical parameter *q*/*p*:


(11) \begin{equation*} {e}^{-k}=\frac{q}{p} \end{equation*}

Estimating infectivity is problematic, especially in humans, because we usually rely on experiments involving human subjects. Such experiments are ethically and scientifically challenging. The gambler’s ruin approach might allow us to estimate ID_50_ and *q*/*p* by surveying populations, particularly for diseases with both apparent and in apparent infections. Suppose that in apparent infections occur in people with ≤ *N* bacteria. The probability of being infected with at least *N* bacteria is:


(12) \begin{equation*} {P}_{\mathrm{infected}}=\left(\frac{1-{\left(\frac{q}{p}\right)}^i}{1-{\left(\frac{q}{p}\right)}^N}\right)\kern1.25em \end{equation*}

If we assume that to be symptomatic you must have >>*N* bacteria, the probability of having symptomatic disease is:


(13) \begin{equation*} {P}_{\mathrm{symptomatic}}=1-{\left(\frac{q}{p}\right)}^i\kern1em \end{equation*}

The fraction of symptomatic infections (*P*_symptomatic_/*P*_infected_) is:


(14) \begin{equation*} \frac{P_{\mathrm{symptomatic}}}{P_{\mathrm{infected}}}=1-{\left(\frac{q}{p}\right)}^i/\left(\frac{1-{\left(\frac{q}{p}\right)}^i}{1-{\left(\frac{q}{p}\right)}^N}\right)\kern1.25em \end{equation*}

If we assume that both the symptomatic and the asymptomatic have received the same dose, then:


(15) \begin{equation*} \frac{P_{\mathrm{symptomatic}}}{P_{\mathrm{infected}}}=1-{\left(\frac{q}{p}\right)}^N \end{equation*}

The ratio of *P*_symptomatic_/*P*_infected_ and the value of *N* can be determined by surveying a population. The value of *q*/*p*, and thus ID_50_ (ID_50_ = ln(0.5)/ln(*q/p*))*,* can, in principle, be determined in a population by conducting quantitative surveys without knowing the dose.

Dizon and colleagues [33] reported that cholera carriers (people with asymptomatic cholera infections) had a geometric mean of 8.53 × 10^3^ vibrios/g in their stools. Assuming the mass of the contents of the large and small intestines is 1200 g [34], this gives a crude estimate of *N* of 7.3 × 10^7^. The ratio of infected-to-symptomatic is purported to be between 1% and 30% [35]. This implies a range of *q/p* values from 0.999999936915 to 0.99999998351 and corresponding ID_50_ values from 1.1 × 10^7^ to 4.4 × 10^7^. This is a tentative and preliminary “back of the envelope” calculation. However, the community ID_50_ estimates are between 10% and 40% of those inferred from healthy prisoner experiments [10]. This could imply that “real-world” infectivity is higher than in healthy adults, that those infected have a slightly higher dose than “carriers”, or that the infected-to-symptomatic ratio is underestimated [35].

### Time to attain a particular abundance

In many situations, we are interested not only in the probability of an outcome but also in how long that outcome will take. In this section, we provide estimates of how presumed or determined *q/p* values, used to calculate the probability of an outcome, can also be used to estimate the time to reach a particular state.

There is an excellent body of literature in this area [21, 36, 37]. However, published works tend to express time in “events”, which, in this context, means births or deaths. What most microbial ecologists wish to know is how many days or years these events will take.

To answer this question, we need a method to convert events (microbial “time”) into solar time (hours or days). The average time for 1 event (a birth or death) depends on the number of bacteria. If 10% of the population undergoes an event per day and there are 1000 bacteria, the mean time between events is 0.001 days; if there are 10^14^ bacteria, the mean time between events is 10^−13^ days. Thus, the mean time between events will increase or decrease as the microbial population increases or decreases.

We must find an equation for the time it takes for a population of a given size to increase or decrease by 1. We then sum those times over the range of abundances of interest, for example from 1 to *n* or from *n* to 0. The relationships depend on whether a population is going up or down, whether *q/p* is or is not equal to one, and the chosen endpoints (the boundary conditions). The derivation of the equations is described in Appendix 3 (for increases) and 4 (for decreases), and the times were compared (Table 1).

**Table 1:** A comparison of the time to a certain abundance or extinction for different ratios of death to birth (*q/p* or *α*).

***q/p* (or *α*)** **Ratio of deaths to births**	**Time to 10** ^**3**^ **(days)**	**Time to 10** ^**6**^ **(days)**	**Time to extinction from 10** ^**3**^ **(days)**	**Time to extinction from 10** ^**6**^ **(days)**
1.01	1.96 × 10^6^	^a^	59	7.03 × 10^3^
1.001	2.13 × 10^3^	^a^	234	8.73 × 10^3^
1.0001	1.30 × 10^3^	5.07 × 10^9^	1.01 × 10^3^	5.94 × 10^4^
1	1.30 × 10^3^	2.68 × 10^4^	6.19 × 10^3^ (carry capacity 10^16^)	4.80 × 10^6^ (carry capacity 10^16^)

a These values could not be calculated by recursion or analytically.

### From 1 to *n*

Let *m_i_* be the additional time that elapses from when a population enters the *i*th abundance class (*i* cells) until it has *i* + 1 cells.


(16) \begin{equation*} {m}_i=\frac{\Delta t}{ip}+\frac{\mathrm{q}}{p}\left({m}_{i-1}\right) \end{equation*}

Equation 16 is a recurrence relationship, because ${m}_i$ depends on ${m}_{i-1}$. We are interested in ${M}_{1,n}$, the expected time to go from a single cell to an abundance, *n*. If there are 0 cells, then there can be no increase in cells, ${m}_0=0$. Equation 16 can be applied recursively to get ${m}_i,\left\{i=1,\dots, n\right\}$ and then since,


(17) \begin{equation*} {M}_{1,n}=\sum_{j=1}^{n-1}{m}_j \end{equation*}

**Figure 5:**
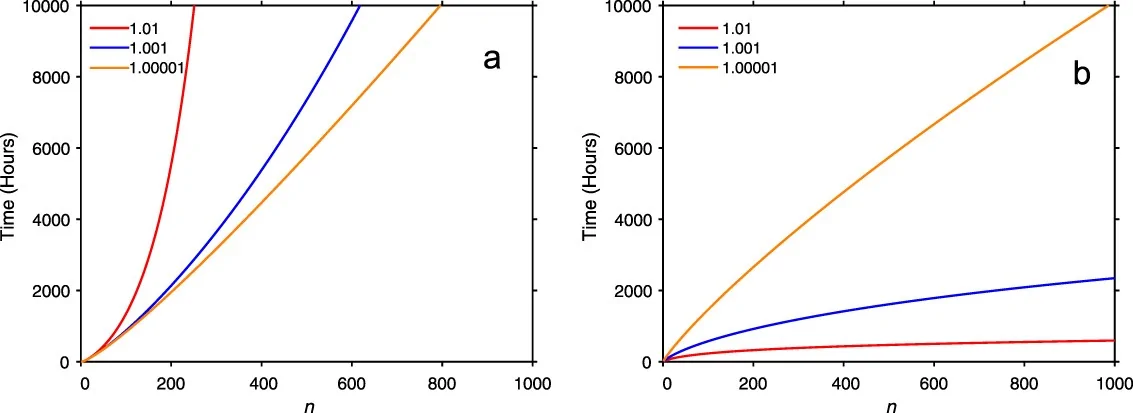
The time for the increase, or extinction, of a population with different values of *q/p* (*α* in the equation). (a) The estimated time for a single organism to attain an abundance of *n*, using equation 18. (b) The time to extinction for an organism with abundance *n*, estimated using equation 27.

It is possible to calculate *M_1,n_* by adding successive values of *m_i_* for values from 1 to *n* on a computer (Figure A1). However, a relatively simple analytical solution is possible (Appendix 3; Figure 5a):


(18) \begin{equation*} {M}_{1,n}=\kern0.5em \frac{\Delta t}{p\left(\alpha -1\right)}\left[{\alpha}^n{S}_{n-1}\left(\alpha \right)-{H}_{n-1}\right] \end{equation*}

where $\alpha\ is\ \frac{\mathrm{q}}{p}$  $, {H}_{n-1}$ is the harmonic number which, for large *n*, can be approximated by,


(19) \begin{equation*} {H}_{n-1}\approx \ln \left(n-1\right)+\gamma +\frac{1}{2\left(n-1\right)}-\frac{1}{12{\left(n-1\right)}^2}+O\left(\frac{1}{{\left(n-3\right)}^3}\right) \end{equation*}

where *γ* = 0.57721 and the sum ${S}_{n-1}\left(\alpha \right)$ is equivalent to the integral,


(20) \begin{equation*} {S}_{n-1}\left(\alpha \right)={\int}_0^{1/\alpha}\frac{1-{t}^{n-1}}{1-t} dt. \end{equation*}

When *α =1*,


(21) \begin{equation*} {m}_i=2\Delta t\sum_{k=1}^n\frac{1}{k}, \end{equation*}

We can use the harmonic number estimate (equation 19) to estimate ${m}_i$ and Stirling’s approximation to find the sum of ${m}_i:$


(22) \begin{equation*} {M}_{1,n}=\sum_{i=1}^{i=n}{m}_i, \end{equation*}

(23) \begin{equation*} =2\Delta t\left(n\ln (n)+n\ \left(-1+\gamma +\frac{1}{2n}-\frac{1}{12{n}^2}\right)\ \right). \end{equation*}

It can take a long time for a single bacterium to grow and produce even a modest-sized population. For example, if *n* = 10^3^ and *Δ t=0.1* days, then ${M}_{1,n}=1.297\times{10}^3$ days and if *n* = 10^6^, then ${M}_{1,n}=2.68\times{10}^6$ days (~7000 years). Simulations showed that random variations in the value of *α* had only a modest effect on these predictions (Figure A3).

### Time from *N* to 0 (extinction)

A simple recursive equation also describes ${\tau}_i$ the time it takes to go from *i* to *i* − 1 (Appendix 4).


(24) \begin{equation*} {\tau}_{i+1}=-\frac{\Delta t}{ip}+\frac{q}{p}{\tau}_i, \end{equation*}

By assuming that *α*>1, we can show that the first term in the series must be


(25) \begin{equation*} {\tau}_1=\kern0.5em \frac{\Delta t}{p{\propto}^{i-1}}\ln \left(1-\frac{1}{\alpha}\right) \end{equation*}

So, the total time to extinction


$$ {T}_i $$

becomes:


(26) \begin{equation*} {T}_i=\sum_{j=1}^{n-1}{\tau}_j. \end{equation*}

Again, it is possible to calculate ${T}_i$ by sampling and adding successive values of ${\tau}_i$ for values from 1 to *n* − 1 on a computer (Figure A2). Or by using a straightforward analytical solution that is derived in a similar way to equation 18 and for which the key terms are already described (Figure 5b):


(27) \begin{equation*} {T}_i=-\frac{\Delta t}{p\left(\alpha -1\right)}\left[{\alpha}^n{S}_{n-1}\left(\alpha \right)-{H}_{n-1}+\left({\alpha}^i-1\right)\ln \left(1-\frac{1}{\alpha}\right)\ \right] \end{equation*}

An alternative approach is required when *α =1*. By assuming the system has a maximum number of bacteria that a population cannot exceed (the carrying capacity) and that the starting abundance is not too small, we show that:


(28) \begin{equation*} {T}_i=2\Delta t\ i\left({H}_{n-1}-{H}_{i-1}\right)+\frac{i}{n}+2\Delta t\left(i-1\right) \end{equation*}

where ${H}_i\approx \ln (i)+\gamma +\frac{1}{2(i)}-\frac{1}{12{(i)}^2}$,

When *α =1* and *Δ t=0.1* days in a system with a carrying capacity of 10^16^, then 10^3^ bacteria will take 17 years to go extinct, and 10^6^ bacteria will take over 13 thousand years (Figure A4).

## Discussion

We have presented a suite of simple equations to estimate the probability and duration of immigration and extinction in microbial communities. We have built on the venerable gambler’s ruin equation, and on the key parameter, the ratio of deaths to births. The ratio of deaths to births has been described as one of the most fundamental parameters in biology [38], yet it is curiously overlooked in microbial ecology. We have shown new ways to estimate this parameter in real communities. The simple and parameterizable nature of the equations will hopefully allow them to be applied to consider immigration, infection, bioaugmentation, extinction, and other related processes. The use of numbers grounded in basic mechanisms and clear endpoints will make success and failure in all these phenomena easier to understand and predict.

All models rest on simplifications. Our most important simplifying assumption is that the ratio of births to deaths is constant. We defend this assumption because it is parsimonious, and because simulations with random variations in the ratio were not markedly different from our “standard” results. Moreover, in pathology, the field in which the ratio of deaths to births was first applied, credible estimates of infectivity were generated, even though the value of *q/p* may vary during an infection [19].

We are not the first to use the gambler’s ruin equation in microbiology. The pioneers in our field certainly [16], or almost certainly [15, 18], knew of this expression. However, they did not report using this equation, perhaps because they were unaware of, or not interested in, scenarios where the probability of a given outcome was not essentially 1. They were interested in the time from infection to the onset of disease [19]. The early models required the user to determine the difference between the probabilities of death and growth; a number so close to zero that it proved difficult to calculate from the animal experiments upon which they relied [18]. The same reliance on the difference between deaths and births is found in subsequent stochastic models of microbial cultures [39, 40]. A study of the emergence of antimicrobial resistance in a Petri dish [41] does propose a synonymous equation for the special case of an inoculum of 1, though the ratio of deaths to births was neither used nor determined.

With one exception [42], other studies of invasion in microbial ecology eschew stochastic models in favour of qualitative assessments or multiple regression [43–45]. In the short run, such approaches are easier for the investigator to conduct. However, in the long term, such models are often situation bound and difficult, or impossible to test. A model, such as ours, grounded in fundamental mechanisms and with clear endpoints permits a more nuanced, generalizable, predictive, and enduring understanding of the field.

The benefits of using a quantitative approach is illustrated by considering our finding that the ratio of deaths to births in stable populations within an established ecosystem (activated sludge) is approximately 1. We admit that a ratio of 1 is intuitively obvious and, given the assumption of stationarity (constant numbers), arithmetically inevitable. However, it presents us with a paradox. In the absence of immigration, a stable population will go extinct [14, 46–48]. Maynard-Smith [25] considered the inevitability of extinction a good reason to avoid stochastic models. We can resolve this paradox by invoking time. Even without immigration, extinction will take thousands of years. Abundance offers some protection against extinction, locally if not globally.

When the ratio of births to deaths is not 1, change can happen quickly, and that rate of change can now be calculated. When the difference between growth and death is large, more conventional approaches may be appropriate [37]. However, in some circumstances, e.g. when considering mutations in a dying population of genetically manipulated organisms, we may need to consider the two processes individually, and our approach may have value for contemporary challenges in engineering biology [49].

Stochastic models invoking immigration in microbial ecology are well established [4]. We have made a link to such models (and reactor kinetics) by showing that the immigration parameter *m* is related to the ratio of deaths to births. This shows, amongst other things, that the value of *m* does not scale with reactor size, a finding we will explore in a subsequent manuscript.

The estimates of the value of *m* are subject to systematic errors. The use of the ratio *X*_inf_*/X*_reactor_ obviates taxon-specific errors due to differential DNA extraction or copy number, but not sample-specific issues in DNA extraction and amplification for amplicons or qPCR. However, it is the ratio of such errors, not the errors themselves, that is problematic. Likewise, omitting “minor” losses due to predation, endogenous decay, or bacterial losses in the effluent could lead to an overestimation of *m.*

Colony-forming organisms could provide insight into the validity of the value of *m* and suggest that the values are correct to within an order of magnitude. For if we assume that a single immigrant forms each colony and that there is no predation within a colony, then the number of cells divided by the number of colonies should be a conservative estimate of *m*. *Nitrosomonas* was the only genus of ammonia-oxidizing bacteria (AOB) detected in this study. AOB typically grow in microcolonies. In a previous study at the same treatment plant [50], the ratio of cells to colonies was 91 (*n* = 277), which is less than the ratio of ~700:1 we estimated. The discrepancy could be explained by systematic errors and deviations from our assumptions about microcolonies (eg, predation and colonies initiated by multiple organisms).

The wide range of *m* values suggests that the notion of dispersal and niche-assembled communities [51] could be discarded in favour of a more nuanced view of immigration as a continuum. However, the curious bimodal distribution of immigration parameters suggests exactly the opposite. Likewise, critics of neutral models can now point out that *m* is a continuum spanning several orders of magnitude. The range of *m* values may be even wider, because some taxa were found in the reactor but not in the influent. However, supporters will point out that the ratio of deaths to births is almost always ~1, supporting neutral theory’s assumption of equal fitness.

We want to highlight some practical implications of our work. A microbial species could go extinct globally and quickly if the ratio of deaths to births is high. Global extinction might occur “by accident” if, e.g. a chemical or class of chemicals were to poison a key enzyme such as ammonia monooxygenase [52]. Where extinction is desirable, e.g. for an antimicrobial resistance gene or a genetically manipulated microorganism (GMMO), we can calculate the probability and duration of such an event [49]. A GMMO with a *q*/*p* ratio of 1.1 is perhaps safer than one with a ratio of 1.00001.

Stable communities are very difficult to invade, even by an organism perfectly adapted to that environment (*q/p* = 1). Thus, the effects of bioaugmentation and probiotics will nearly always be transitory and will only be effective where the organism can have the desired effect before being driven to extinction. Often, it will be easier to change the environment to ensure *q/p* < 1 (e.g. by adding a nutrient [53]) than to design an organism with this property. Indeed, a change in environment seems to permit successful invasion in nature [54] and in engineering [55]. The ratio of death to birth is more likely to be <1 when a community is starting up. Consequently, seeding could be a better strategy for incorporating taxa into a community. We speculate that faecal transplantation often works because (amongst other things) relatively large numbers of immigrants are used, and the value of *q*/*p*, which in a stable community would be ~1, is reduced when the host is treated with antibiotics [9].

It may be possible to infer infectivity simply and ethically (where *q*/*p* is <1) by observing naturally infected populations. Our crude calculation of ID_50_ and *q*/*p* for *V. cholerae* supports this proposal. Further work, more thought, and better data and statistics are required before we can be more definitive about this approach. More realistic dose–response curves might be helpful in quantitative microbial risk assessments. The assumption that the dose is the same in both symptomatic and asymptomatic subjects is conservative. That is, it could lead us to overestimate infectivity and, in turn, overstate the risk of infection. Even if it is wrong, it is safe.

## Supplementary Material

Web_Material_wrag195

## Data Availability

The raw sequence data for this study have been deposited in the European Nucleotide Archive (ENA) at EMBL-EBI under accession number PRJEB121522. In addition, relevant code (including the code for estimating the duration of immigration and extinction) is available at https://github.com/beadyallen/PPD_Immigration_Microbe. A database of the values of *m* and *q/p* are also presented in the supplementary material.
