# Supplementary material for: Probability, parameters, and duration of immigration and extinction in microbial communities
Source: ISME J. 2026 Jul 22;20(1):wrag195. doi: 10.1093/ismejo/wrag195 (PMC13575278; doi:10.1093/ismejo/wrag195)
Supplement: Supplementary file 1 [file web_material_wrag195.zip › Appendices V22b.pdf]

Appendix 1: Derivation of the Gamblers Ruin Problem [1] (Page 213).

In our idealised community a single bacterium will increase with the probability p (λ in classical texts) and die with probability q (μ in classical texts) where q=1-p.

Let P_i be the probability that an inoculum of size i will attain abundance N and i can be any number from 1 to N-1. Then,

$$P_i = pP_{i+1} + qP_{i-1} \, ,$$

but p+q= 1, so,

$$pP_i + qP_i = pP_{i+1} + qP_{i-1} \, ,$$

$$P_{i+1} - P_i = \frac{q}{p}(P_i - P_{i-1}) \, .$$

When i = 1, P_{i-1} =0 and, therefore,

$$P_2 - P_1 = \frac{q}{p}P_1 \, ,$$

$$P_3 - P_2 = \frac{q}{p}(P_2 - P_1) \, ,$$

$$P_3 - P_2 = \frac{q}{p}\left(\frac{p}{q}P_1 \right) ,$$

$$P_3 - P_2 = \left(\frac{q}{p}\right)^2 P_1 \, ,$$

$$P_i - P_{i-1} = \left(\frac{q}{p}\right)^{i-1} P_1 \, .$$

Adding i-1 terms allow us to calculate,

$$P_i - P_1 = P_1\left[\left(\frac{q}{p}\right) + \left(\frac{q}{p}\right)^2 +..+ \left(\frac{q}{p}\right)^{i-1}\right] \, ,$$

or,

$$P_i = P_1\left[1 + \left(\frac{q}{p}\right) + \left(\frac{q}{p}\right)^2 +..+ \left(\frac{q}{p}\right)^{i-1}\right]$$

The terms inside the square bracket are the sum of a geometric series so,

$$P_i = \begin{cases} \frac{1-(q/p)^i}{1-(q/p)} P_1 \, , & \text{if } \frac{q}{p} \neq 1 \, ; \\ iP_1 \, , & \text{if } p = q = 0.5 \, . \end{cases} \qquad (1.1)$$

P_N is the probability of N bacteria attaining an abundance of N, which is obviously 1. Thus, if P_N =1 we can rearrange to calculate P_1 , the probability of a single new bacterium attaining a density of N.

$$1 = P_N = \begin{cases} \frac{1-(q/p)^N}{1-(q/p)} P_1 \, , & \text{if } \frac{q}{p} \neq 1 \, ; \\ NP_1 \, , & \text{if } p = q = 0.5 \, , \end{cases} \qquad (1.2)$$

and so,

$$P_1 = \begin{cases} \frac{1-(^q/_p)}{1-(^q/_p)^N}, & \text{if } \frac{q}{p} \neq 1\ ; \\ \frac{1}{N}, & \text{if } p = q = 0.5\ . \end{cases} \tag{1.3}$$

This represents the probability of single new organism, for example a new mutation, acquiring an abundance of N.

We can determine P_i, the probability of *i* organisms attaining an abundance *N* if we substitute equation 1.1 by equation 1.3,

$$P_i = \begin{cases} \frac{1-(^q/_p)^i}{1-(^q/_p)^N}, & \text{if } \frac{q}{p} \neq 1\ ; \\ \frac{i}{N}, & \text{if } p = q = 0.5\ . \end{cases} \tag{1.4}$$

Thus, the probability of an organism with a $q/p > 1$ attaining an abundance of *N* is very low indeed even if *N* and *i* are very close.

Appendix 2: Immigration in reactors

If $X_{reactor}$ is the concentration of cells (cells/m^3) of particular species of bacteria in the mixed liquor in a reactor, X_{inf} is their concentration in influent, *Q* is the flow (m^3/day), μ is the growth rate (d^{-1}), *V* is the volume (m^3) and θ is the fraction of bacteria dying per day due to sludge wastage and other causes (d^{-1}), then

$$\frac{dX_{reactor}V}{dt} = X_{inf}Q + \mu X_{reactor}V - \theta X_{reactor}V\ . \tag{2.1}$$

At steady state,

$$0 = X_{inf}Q + \mu X_{reactor}V - \theta X_{reactor}V\ . \tag{2.2}$$

Because we are at equilibrium, the deaths are replaced by either growth or immigration. This equilibrium assumption has been shown to be true over long time periods.

Rearranging,

$$\theta X_{reactor}V = X_{inf}Q + \mu X_{reactor}V\ . \tag{2.3}$$

Now the term QX_{inf} is the biomass of the species flowing into the reactor per day, but it is not the immigration rate, m, because immigration is expressed per death and the number of deaths is $\theta X_{reactor}V$. So, dividing through by the death rate,

$$\frac{\theta X_{reactor}V}{\theta X_{reactor}V} = \frac{X_{inf}Q}{\theta X_{reactor}V} + \frac{\mu X_{reactor}V}{\theta X_{reactor}V}, \tag{2.4}$$

and simplifying, gives,

$$1 = \frac{X_{inf}Q}{\theta X_{reactor}V} + \frac{\mu}{\theta}\ . \tag{2.5}$$

This term only applies where the growth rate $\mu \geq 0$, for otherwise μ is a death term and should be in the denominator.

The hydraulic retention time HRT (days) is *V/Q* so,

$$1 = \frac{X_{inf}}{\theta X_{reactor} HRT} + \frac{\mu}{\theta} . \quad (2.6)$$

The parameter m is number of immigrations per death so, at steady state,

$$m = 1 - \frac{\mu}{\theta} = \frac{X_i}{\theta X_{reactor} HRT} . \quad (2.7)$$

We now have relationship between the equilibrium value for the immigration parameter *m* the ratio births deaths $\frac{\mu}{\theta}$. The ratio $\frac{\theta}{\mu}$ is an estimate of $\frac{q}{p}$, so

$$\frac{q}{p} \sim \frac{\theta}{\mu} = \frac{1}{1-m} . \quad (2.8)$$

Appendix 3: Time to *n*.

Standard texts (Ross, 2003) derive the expected number of events required to get from an abundance *i* to abundance N. Here we are interested in the expected times to attain particular abundances, rather than the number of events. It is important to appreciate that the expected time required for a cell to subdivide is Δt, then as the population increases the expected time between births and deaths in the population decreases proportionately.

Let $M_{i,n}$ is the expected time required to get from state i cells to state n cells. Then

$$M_{i,n} = \frac{\Delta t}{i} + pM_{i+1,n} + qM_{i-1,n} ,$$

and so,

$$p\left(M_{i,n} - M_{i+1,n}\right) = \frac{\Delta t}{i} + q(M_{i-1,n} - M_{i,n}) . \quad (3.1)$$

Now let $m_i = M_{i,n} - M_{i+1,n}$, which is the expected time required to go from abundance i $i+1$ such that,

$$M_{i,n} = \sum_{j=i}^{n-1} m_j . \quad (3.2)$$

Substituting in to (3.1) gives,

$$m_i = \frac{\Delta t}{ip} + \frac{q}{p}(m_{i-1}) . \quad (3.3)$$

This recursive relationship and the sum (3.2) along with an appropriate boundary condition provide and easy and efficient means of estimating the expected time, $M_{i,n}$.

We are particularly interested in $M_{1,n}$, the expected time to go from a single cell to an abundance, n. Since $m_0 = 0$, equation (3.3) can be applied recursively to get $m_i, \{i = 1, \ldots, n\}$ and then,

$$M_{1,n} = \sum_{j=1}^{n-1} m_j . \quad (3.4)$$

It is also possible to derive analytical expressions of the expected times. So, again, consider $M_{1,n}$. Recursively applying equation (3.3) to calculate m_1(since $m_0 = 0$), gives,

$$m_1 = \frac{\Delta t}{1p},$$

$$m_2 = \frac{\Delta t}{2p} + \frac{\mathrm{q}}{p}\frac{\Delta t}{1p},$$

$$\begin{aligned} m_3 &= \frac{\Delta t}{3p} + \frac{\mathrm{q}}{p}\left(\frac{\Delta t}{2p} + \frac{\mathrm{q}}{p}\frac{\Delta t}{1p}\right) \\ &= \frac{\Delta t}{3p} + \frac{\Delta t q}{2p^2} + \frac{\Delta t q^2}{1\,p^3} \\ &= \frac{\Delta t}{p}\left(\frac{1}{3} + \frac{\mathrm{q}}{2p} + \frac{q^2}{1p^2}\right). \end{aligned}$$

Letting $\propto = \frac{q}{p}$, then it is apparent that,

$$m_i = \frac{\Delta t}{p}\left(\frac{1}{i}\alpha^{i-i} + \frac{1}{i-1}\alpha^{i-(i-1)} + \cdots \frac{1}{1}\alpha^{i-1}\right),$$

or,

$$m_i = \frac{\Delta t}{p}\sum_{j=1}^{i}\frac{\alpha^{i-j}}{j}. \tag{3.5}$$

So,

$$M_{i,n} = \frac{\Delta t}{p}\sum_{j=i}^{n-1}\sum_{k=1}^{j}\frac{\alpha^{j-k}}{k},$$

and

$$M_{1,n} = \frac{\Delta t}{p}\sum_{j=1}^{n-1}\sum_{k=1}^{j}\frac{\alpha^{j-k}}{k}.$$

Rearranging the summation,

$$\begin{aligned} M_{1,n} &= \frac{\Delta t}{p}\sum_{k=1}^{n-1}\sum_{j=k}^{n-1}\frac{\alpha^{j-k}}{k} \\ &= \frac{\Delta t}{p}\sum_{k=1}^{n-1}\frac{1}{k}\sum_{j=k}^{n-1}\alpha^{j-k}. \end{aligned}$$

let $m = j - k$ then

$$M_{1,n} = \frac{\Delta t}{p}\sum_{k=1}^{n-1}\frac{1}{k}\sum_{m=0}^{n-1-k}\alpha^{m},$$

and, therefore,

$$\begin{aligned} M_{1,n} &= \frac{\Delta t}{p}\sum_{k=1}^{n-1}\frac{1}{k}\sum_{m=0}^{n-1-k}\alpha^{m} \\ &= \frac{\Delta t}{p}\sum_{k=1}^{n-1}\frac{1}{k}\left(\frac{\alpha^{n-k}-1}{\alpha-1}\right) \\ &= \frac{\Delta t}{p(\alpha-1)}\sum_{k=1}^{n-1}\frac{\alpha^{n-k}}{k} - \sum_{k=1}^{n-1}\frac{1}{k} \\ &= \frac{\Delta t}{p(\alpha-1)}\left[\alpha^{n}\sum_{k=1}^{n-1}\frac{\alpha^{-k}}{k} - \sum_{k=1}^{n-1}\frac{1}{k}\right] \\ &= \frac{\Delta t}{p(\alpha-1)}\left[\alpha^{n}S_{n-1}(\alpha) - H_{n-1}\right]. \end{aligned} \tag{3.6}$$

where H_{n-1} is the harmonic number which, for large n, can be approximated by,

$$H_{n-1} \approx \ln(n-1) + \gamma + \frac{1}{2(n-1)} - \frac{1}{12(n-1)^2} + O\left(\frac{1}{(n-3)^3}\right) \quad , \qquad (3.7)$$

where γ = 0.57721 and the sum $S_{n-1}(\alpha)$ is equivalent to the integral,

$$S_{n-1}(\alpha) = \int_0^{1/\alpha} \frac{1-t^{n-1}}{1-t} dt. \qquad (3.8)$$

Equation 3.8 is a standard finding in the mathematics of series and *t* is a dummy variable, not time. The big *O*( ) notation in equation 3.7 gives an indication of the error in the approximation and, in this case, states that it is no larger than a constant time $(n-3)^3$.

A comparison between the recurrence method (Equations 3.3 -3.4) and the analytic solution (Equation 3.6) for estimating the time to go from 1 bacterium to *n* found no difference between the for n up to 1000 (Figure A1).

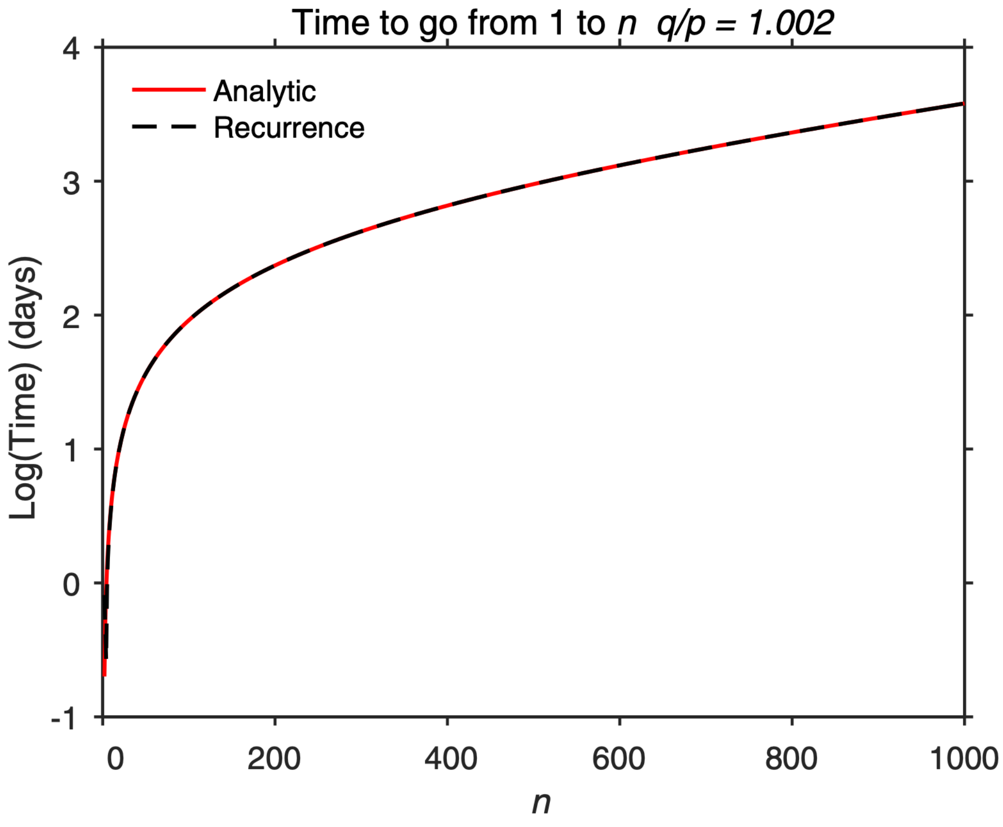

Figure A1. Comparison of the two methods of calculating the time to go from a single bacterium to *n*.

Now consider the special case of $\alpha = 1$, then

$$m_i = 2\Delta t \sum_{k=1}^{n} \frac{1}{k} \ ,$$

which can be approximated using (Equation 3.7) as,

$$m_i = 2\Delta t\left(\ln(i) + \gamma + \frac{1}{2(i)} - \frac{1}{12(i)^2}\right).$$

Therefore, using Stirling's approximation

$$M_{1,n} = \sum_{i=1}^{i=n} M_i\ ,$$

$$= 2\Delta t\left(n\ln(n) + n\ \left(-1 + \ \gamma + \frac{1}{2n} - \frac{1}{12n^2}\right)\right). \qquad (3.9)$$

Thus it can take a long time for a single bacterium to grow and produce even a modest size population, For example, if n = 10^3 and $\Delta t = 0.1$ days , then $M_{1,n} = 1.297 \times 10^3$ days and if n = 10^6 , then $M_{1,n} = 2.68 \times 10^6$ days.

Appendix 4. Time to extinction, starting from *i* .

The time to go extinct can be calculated in a very similar manner to the time to go from *i* to *n* in Appendix 3. We let T_i be the expected time required to get from state *i* cells to state 0 cells. Then,

$$T_i = \frac{\Delta t}{i} + pT_{i+1} + qT_{i-1}\ . \qquad (4.1)$$

Rearranging,

$$p(T_i - T_{i+1}) \ = \frac{\Delta t}{i} + q(T_{i-1} - T_i).$$

Now let $\tau_{i+1} = \ T_{i+1} - T_i$ be the time expected to drop to *i* from *i*+1, which is the reverse of the event in appendix 3, then,

$$\tau_{i+1} = -\frac{\Delta t}{ip} + \frac{q}{p}\tau_i\ , \qquad (4.2)$$

and

$$T_i = \ \sum_{j=1}^{i}\tau_j. \qquad (4.3)$$

In this case $\tau_1 \neq 0$, instead we need to ensure that the time to extinction is finite. So, that, letting $\propto = \frac{q}{p}$,

$$\tau_2 = \ -\frac{\Delta t}{p} + \alpha\tau_1\ , \qquad (4.4)$$

$$\tau_3 = \ -\frac{\Delta t}{2p} + \alpha\left(-\frac{\Delta t}{ip} + \alpha\tau_1\right). \qquad (4.5)$$

So,

$$\tau_i = \ \tau_1 \propto^{i-1} - \frac{\Delta t}{p}\sum_{j=1}^{i-1}\frac{\alpha^{i-1-j}}{j}\ . \qquad (4.6)$$

Now for T_i to stay bounded as $i\ \to\ \infty$ then $\tau_i\ \to\ 0$ and, therefore,

$$0 = \propto^{i-1}\ \left(\tau_1 - \frac{\Delta t}{p}\sum_{j=1}^{\infty}\frac{\alpha^{-j}}{j}\right),$$

and so, assuming $\propto > 1$,

$$\tau_1 = \frac{\Delta t}{p\propto^{i-1}} \sum_{j=1}^{\infty} \frac{\alpha^{-j}}{j}$$
$$= \frac{\Delta t}{p\propto^{i-1}} \ln\left(1 - \frac{1}{\alpha}\right) . \qquad (4.7)$$

Now Equations 4.2, 4.3 and 4.7 can be used to determine T_i recursively.

Alternatively, we can derive an analytical form in a similar way to appendix 3,

$$T_i = -\frac{\Delta t}{p(\alpha-1)} \left[\alpha^n S_i(\alpha) - H_i + (\alpha^i - 1)\ln\left(1 - \frac{1}{\alpha}\right)\right] . \qquad (4.8)$$

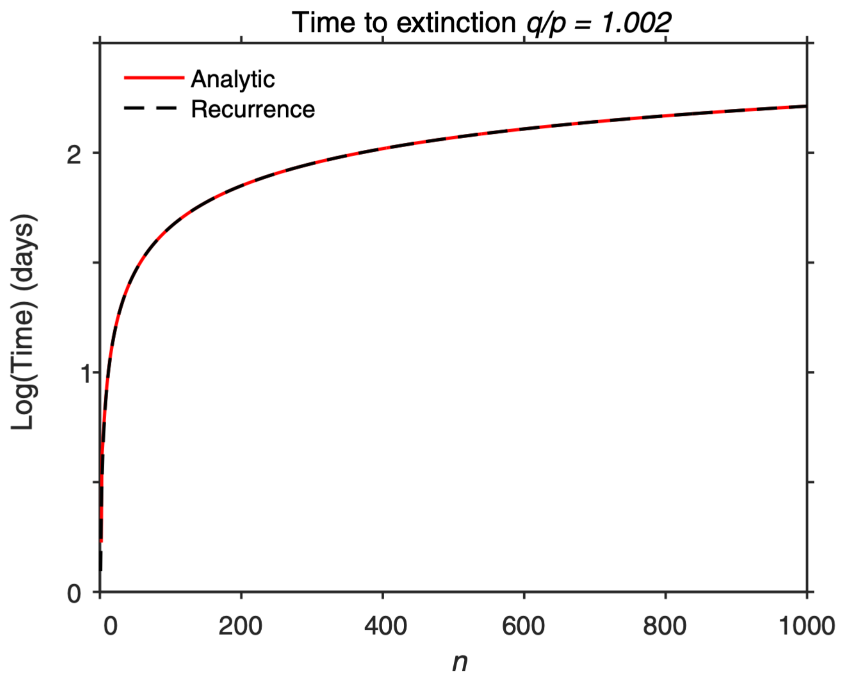

Figure A2. Comparison of the two methods of calculating the time to go from *n* to 0.

Both the analytic and recurrence estimated of the time to go extinct with and without noise in the value of the ratio. The effect of noise increases with increasing n (Figure A3 and A4).

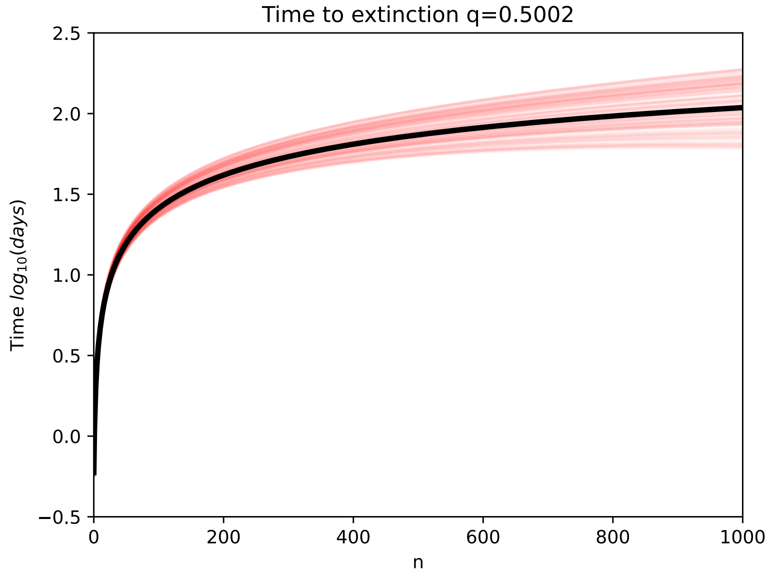

Figure A3 Effect of random noise on time to extinction from state n. Random uniform noise trajectories (red lines, absolute magnitude +- 2e-4) added to base q=0.5002 (solid black line). Calculated using recurrence relationship equation 4.1.

4.1 Extinction when $\propto = 1$

In the derivation above we assumed that $\propto > 1$ and so the sum $\sum_{j=1}^{\infty} \frac{\alpha^{-j}}{j}$ was finite and given by $ln\left(1 - \frac{1}{\alpha}\right)$. Unfortunately, when $\propto = 1$ we get $\sum_{j=1}^{\infty} \frac{1}{j}$, which is infinite. Therefore, when *p* = *q* = 0.5 the time to extinction becomes infinite. However, if we assume that there is some threshold in abundance that the species cannot exceed, then we can calculate the expected time to extinction. The idea of bacteria having a limiting maximum attainable abundance in a particular environment is embedded in many microbial population dynamic models and is often referred to as the carry capacity. If we substitute $\propto = 1$ into Equation 4.6 then we get,

$$\tau_i = \tau_1 - 2\Delta t \sum_{j=1}^{i-1} \frac{1}{j} . \tag{4.9}$$

Now we let the carry capacity be *n* and if the abundance gets to *n* then it can only step down to *n*-1 so,

$$\tau_n = \frac{1}{n} = \tau_1 - 2\Delta t \sum_{j=1}^{n-1} \frac{1}{j}. \tag{4.10}$$

So,

$$\tau_1 = \frac{1}{n} + 2\Delta t \sum_{j=1}^{n-1} \frac{1}{j} , \quad (4.10)$$

and,

$$\tau_i = \frac{1}{n} + 2\Delta t \sum_{j=1}^{n-1} \frac{1}{j} - 2\Delta t \sum_{j=1}^{i-1} \frac{1}{j} . \tag{4.11}$$

Or, using the harmonic number notation,

$$\tau_i = \frac{1}{n} + 2\Delta t H_{n-1} - 2\Delta t H_{i-1} . \tag{4.12}$$

Now,

$$T_i = \sum_{j=1}^{i} \tau_j \tag{4.13}$$

$$= i\left(\frac{1}{n} + 2\Delta t H_{n-1}\right) - 2\Delta t \sum_{j=1}^{i} H_{j-1} \tag{4.14}$$

It can be shown that,

$$\sum_{j=1}^{i} H_{j-1} = iH_{i-1} - (i-1), \tag{4.15}$$

and so,

$$T_i = i\left(\frac{1}{n} + 2\Delta t H_{n-1}\right) - 2\Delta t(iH_{i-1} - (i-1)) \tag{4.16}$$

$$T_i = 2\Delta t\, i(H_{n-1} - H_{i-1}) + \frac{i}{n} + 2\Delta t(i-1) \ . \tag{4.17}$$

Both the analytic and recurrence estimated of the time to go extinct when $\propto = 1$ with different carrying capacities were compared (Figure A4) . Note that here we have approximated the harmonic numbers $H_{n-1}$and H_{i-1} using,

$$H_i \approx \ln(i) + \gamma + \frac{1}{2(i)} - \frac{1}{12(i)^2} \ , \tag{4.18}$$

which introduces errors. In the case of the recurrence relationship the error is introduced $T_1$and propagates, thus growing as *i* increases, this explains the discrepancies between the analytic and recursive solutions (Figure A4). The approximation Equation 4.18 is more accurate for large n. Therefore, for small carrying capacities, *n,* it is better to fully evaluate the harmonic number.

It is also worth noting that the time to extinction for a given *i* scales proportional to $\ln\left(\frac{n}{i}\right)$ and so whilst the unbounded process described in appendix 3 has an infinite time to extinction, even if the carry capacity was 10^{30}, the number of bacteria estimated to be on the earth, the extinction time is less than $2\Delta t$ x71*i*.

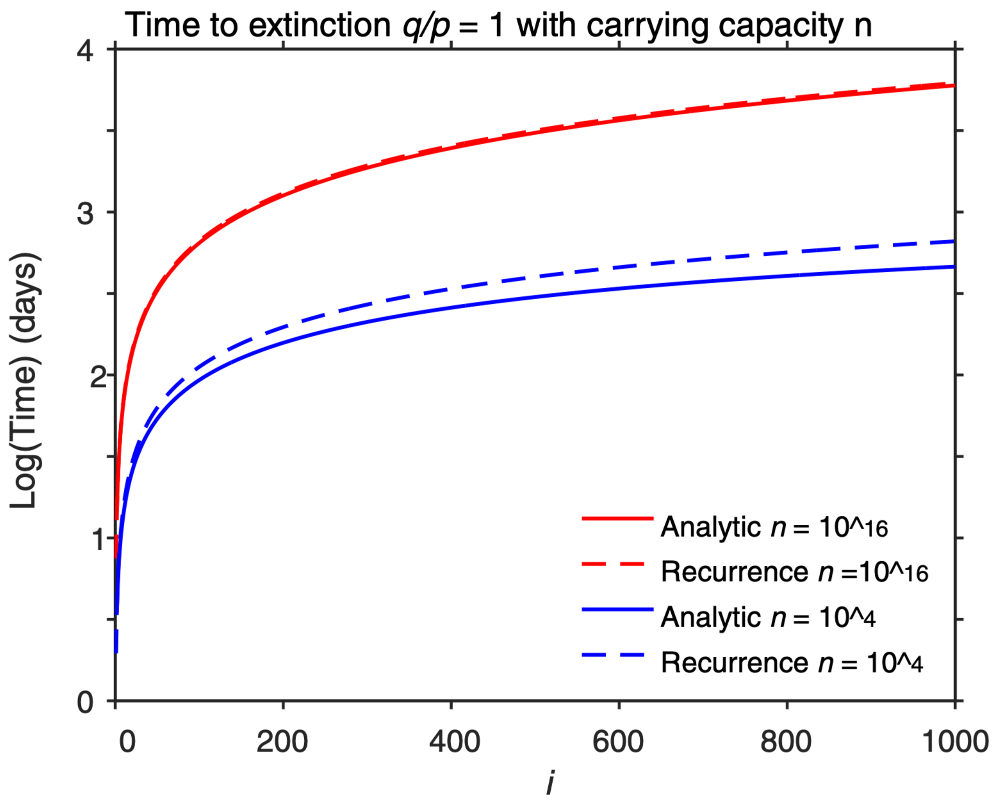

Figure A4. Time to go extinct starting at i for different carrying capacities.

Appendix 5. Methods

The collection and analysis of influent and effluent wastewater has been reported previously [2].

Grab samples were collected from the mixed liquor in the aeration basin (3600 m^3) and the settled sewage (the influent for the aeration basin) of a conventional nitrifying domestic wastewater treatment plant (situated in the North East of England, United Kingdom, on a weekly basis for a period of five years from June 2011 to June 2016 (258 sampling events). The flow and solids retention time were also noted.

DNA extraction

DNA was extracted from 250 µL of ML and from 15 mL of influent, the latter being centrifuged at 3392×*g* for 15 min and the supernatant removed down to a working volume of 250 µL. Cell wall disruption was then carried out using the FastDNA SPIN Kit for soil (MP Biomedicals, USA), thus 244.5 µL of sodium phosphate buffer and 30.5 µL of MT buffer was added to samples and the mixture transferred to Lysing Matrix E tubes. Samples were then lysed at 6.5 ms^{-1} for 30 s in a FastPrep instrument (MP Biomedicals, USA) and centrifuged at 14000×*g* for 15 min. DNA from 250 µL of the supernatant was then purified using a MagNA Pure LC 2.0 (Roche, UK) and the MagNA Pure LC DNA Isolation Kit S.

qPCR

Quantification of total bacteria was carried out using qPCR and amplification of the 16S rRNA gene. Samples were amplified in triplicate on a CFX96 Real-Time PCR Detection System (Bio-Rad, UK) using the primer sets 338F [3] and 1046R [4]. qPCR amplifications contained 3 µL of template DNA (sample DNA, standard DNA, or molecular grade water (negative control)), 0.5 µL of forward and reverse primer (10 ρmoles per µL), 5 µl of SsoFast EvaGreen supermix (Bio-Rad, UK) and 1 µl of molecular-grade water. Reaction conditions were: 1 cycle at 98 °C for 3 min, followed by 40 cycles consisting of 98 °C for 5 s and 60 °C. Purified circular plasmids containing the target gene were used as standards and run in triplicate for each qPCR amplification. Efficiencies for all qPCR amplifications ranged between 90 and 110% and had a R^2 ≥ 0.99.

Illumina sequencing

Sample preparation for sequencing generally followed the protocol of Caporaso et al. [5]. Thus, the V4 region of the bacterial 16S rRNA gene was amplified using primers 515F (aatgatacggcgaccaccgagatctacactatggtattgtGTGCCAGCMGCCGCGGTAA; adapter, primer pad and primer linker in small letters; and specific primer sequence in capital letters) and 806R (caagcagaagacggcatacgagat**barcode**agtcagtcagccGGACTACHVGGGTWTCTAAT), the latter was barcoded with a 12-base error-correcting Golay code to facilitate sample multiplexing. Each sample was amplified in duplicate, pooled and then cleaned using a MinElute 96 UF Purification Kit as per the manufacturer's instructions (Qiagen Ltd., West Sussex, UK). PCR amplifications consisted of: 2 µl DNA extract, 20 µl 5 Prime Hot Master Mix (VWR, Lutterworth, UK), 1 µl each of forward and reverse primer (10 µM final concentration), and 26 µl molecular-grade water. Reactions were denatured at 94 °C for 3 min, with amplification for 35 cycles at 94 °C for 45 s (further denaturing), 50 °C for 60 s (annealing) and 72 °C for 90 s (extension); a final extension at 72 °C for 10 min was added to ensure complete amplification. The concentration and purity of pooled amplicons was assessed using a Quant-iT PicoGreen dsDNA assay kit (Life , Paisley, UK). A composite sample for sequencing was created by combining all samples in equimolar amounts. The composite sample was cleaned twice using Agencourt AMPure XP beads (Beckman Coulter, High Wycombe, UK) according to the manufacturer's instructions, with fragment selection undertaken using E-Gel 2% agarose gels (Life Technologies, Paisley, UK). The size-selected fragment was then purified using the QIAquick PCR purification kit (Qiagen Ltd., West Sussex, UK) and quantified using the Quant-iT PicoGreen dsDNA assay kit, to ensure the sample contained enough DNA for sequencing analysis. Sequencing was carried out on the MiSeq System (Illumina, San Diego, Ca), at the Centre for Genomic Research University of Liverpool.

Raw reads were reprocessed for this work using the DADA2 (v. 1.26, [6]) pipeline, specifically following the workflow for Big Data (https://benjjneb.github.io/dada2/bigdata.html), and using R version 3.4.0, [7]). Forward and reverse read pairs were trimmed and filtered (minimum length 200 nucleotides, EEmax < 2 expected errors). Amplicon sequence variants (ASV's) were independently inferred in each sample from forward and reverse reads using the run-specific error rates, and then joined using the ~mergePairs ~ function. Chimeric ASVs were inferred and identified using ~ removeBimeraDenovo~ and removed. This final ASVs (per sample were taxonomically classified against the SILVA database (v.138.2, [8]) using the DADA2 implementation of the RDP's naive Bayesian classifier [9].

ASV absolute abundances per sample were calculated by multiplying ASV relative abundances by accompanying total bacteria numbers from the qPCR data.

Estimates of the Errors in the Ratio of influent to effluent biomass.

We have estimated the errors in the estimate of the ratio of taxa abundances in the influent and effluent using the methods and notations of [10, 11].

We have estimated x_i the abundance of a specific organism by multiplying the proportional abundance p_i in n samples by a total abundance estimated using QPCR X_T for the same n samples,

$$x_i = p_i X_T. \tag{5.1}$$

Mclaren et al., have identified three sources of error: those that are specific to an organism i (B_i), the weighted mean efficiency for all taxa $\widetilde{B}$, and the mean efficiency of the total-abundance measurement B_T.

We have made measurements of x_i that are subject to error,

$$\tilde{x}_i = \tilde{p}_i \tilde{X}_T \ , \tag{5.2}$$

where the tilde is the measurement subject to error(s). The error affected X_T is,

$$\tilde{X}_T = X_T B_T \ , \tag{5.3}$$

where B_T is the error of the method, in this case QPCR, and X_T is the true value of X_T ,

$$\tilde{p}_i = p_i \frac{B_i}{\tilde{B}}, \tag{5.4}$$

where B_i is the taxon specific error, $\tilde{B}$ is the average taxon specific error in a milieu, and p_i is the true value of p_i.

So, the overall term is,

$$\tilde{x}_i = p_i \frac{B_i}{\tilde{B}} X_T B_T, \tag{5.5}$$

However, we are interested in the ratio between the bacteria in the influent (indicated by an extra subscript I; $\tilde{x}_{i,i,} p_{i,i}\ X_{T,i}\ \widetilde{B}_i\ B_{T,i}$) and the bacteria in the reactors (indicated by an extra subscript r; $\tilde{x}_{i,r} p_{i,r}\ X_{T,r}\ \widetilde{B}_r\ B_{T,r}$).

So, the ratio of measurements and errors in our method is

$$\frac{\tilde{x}_{i,i}}{\tilde{x}_{i,r}} = \frac{p_{i,i}\, X_{T,i}\, \widetilde{B}_r\, B_{T,i}}{p_{i,r}\, X_{T,r}\, \widetilde{B}_i\ \ B_{T,r}}, \tag{5.6}$$

So, the error term $\frac{\widetilde{B}_r\, B_{T,i}}{\widetilde{B}_i\ \ B_{T,r}}$ is constant for all taxa and a function of a ratio of the sample specific errors.

References

1. Ross S. *Introduction to probability models*, San Diego: Academic Press, 2003.
2. Brown MR, Baptista JC, Lunn M *et al.* Coupled virus - bacteria interactions and ecosystem function in an engineered microbial system. *Water Res*. 2019;**152**:264-73 https://doi.org/10.1016/j.watres.2019.01.003
3. Muyzer G, Dewaal EC, Uitterlinden AG. Profiling of complex microbial-populations by denaturing gradient gel-electrophoresis analysis of polymerase chain reaction-amplified genes-coding for 16S ribosomal-RNA. *Appl Environ Microbiol*. 1993;**59**:695-700 https://doi.org/10.1128/aem.59.3.695-700.1993.
4. Huber JA, Welch DBM, Morrison HG *et al.* Microbial population structures in the deep marine biosphere. *Science*. 2007;**318**:97-100
5. Caporaso JG, Lauber CL, Walters WA *et al.* Ultra-high-throughput microbial community analysis on the illumina hiseq and miseq platforms. *ISME J*. 2012;**6**:1621-4 https://doi.org/10.1038/ismej.2012.8
6. Callahan BJ, McMurdie PJ, Rosen MJ *et al.* DADA2: High-resolution sample inference from illumina amplicon data. *Nat Methods*. 2016;**13**:581-3 https://doi.org/10.1038/nmeth.3869
7. Team R. *A language and environment for statistical computing*, Vienna, Austria: R Foundation for Statistical Computing, 2017.
8. Quast C, Pruesse E, Yilmaz P *et al.* The silva ribosomal RNA gene database project: Improved data processing and web-based tools. *Nucleic Acids Res*. 2013;**41**:D590-D96 https://doi.org/10.1093/nar/gks1219
9. Wang Q, Garrity GM, Tiedje JM *et al.* Naive bayesian classifier for rapid assignment of rRNA sequences into the new bacterial taxonomy. *Appl Environ Microbiol*. 2007;**73**:5261-7 https://doi.org/10.1128/aem.00062-07
10. McLaren MR, Willis AD, Callahan BJ. Consistent and correctable bias in metagenomic sequencing experiments. *eLife*. 2019;**8**:e46923 https://doi.org/10.7554/eLife.46923
11. McLaren MR, Nearing JT, Willis AD *et al.* Implications of taxonomic bias for microbial differential-abundance analysis. https://mikemc.github.io/differential-abundance-theory/v/7412a36ddb8cad3c1c0f3ff2055f5402c4a74360/case-studies.html#microbial-growth-in-marine-sediments (21 December 2025, date last accessed).
